# Associations of Omega-3 and Omega-6 Fatty Acids Intake with Visceral Adiposity: Sex-Specific Patterns in a Population-Based Study

**DOI:** 10.3390/molecules30214245

**Published:** 2025-10-31

**Authors:** Livia Alvarenga, Ribanna A. M. Braga, Júlia G. de Souza, Julia T. Y. Iorio, Luciane Coutinho de Azevedo, Ernani T. de Santa Helena, Nágila R. T. Damasceno

**Affiliations:** 1 Department of Cardiopneumology, Faculty of Medicine, University of São Paulo, São Paulo 01246-903, SP, Brazil; liviaalvarenga@usp.br; 2 Postgraduate Program in Cardiology, Heart Institute (InCor), Faculty of Medicine, University of São Paulo, São Paulo 05403-900, SP, Brazil; ribanna.marques@usp.br (R.A.M.B.); juliagalbiati@usp.br (J.G.d.S.); 3 Department of Nutrition, Faculty of Public Health, University of São Paulo, São Paulo 01246-904, SP, Brazil; tiekojulia@usp.br; 4 Postgraduate Program in Public Health, University of Blumenau, Blumenau 89030-900, SC, Brazil; lucianec@furb.br (L.C.d.A.); erntsh@furb.br (E.T.d.S.H.)

**Keywords:** visceral adiposity index, omega-3, EPA, DHA, polyunsaturated fatty acids

## Abstract

Background: Obesity is a disease with high prevalence worldwide, and the accumulation of visceral fat is related to increased cardiovascular risk. The inclusion of polyunsaturated fatty acids (PUFAs) in the diet has been shown to be a promising nutritional strategy. We aimed to examine the associations between PUFAs consumption and visceral adiposity dysfunction, assessed by the visceral adiposity index (VAI). Methods: This cross-sectional study included 697 adults from the SHIP-Brazil cohort. Structured interviews collected sociodemographic, lifestyle, and dietary data. The intake of omega-3, omega-6, EPA, DHA, and EPA + DHA intake assessed through an adapted food frequency questionnaire (FFQ) was categorized into tertiles. Serum lipids were analyzed using the Cobas system, and VAI was calculated by a sex-specific formula and categorized into two groups (low and high VAI, p50), according to sex. Results: Among men, a higher VAI was associated with greater energy intake and higher carbohydrate and fat consumption. Among women, EPA + DHA intake (β = −0.396, 95% CI: −0.639; −0.152, *p* = 0.001), EPA (β = −0.679, 95% CI: −1.220; −0.138, *p* = 0.014), and DHA (β = −0.780, 95% CI: −1.207; −0.352, *p* < 0.001) were negatively associated with VAI, while omega-6 (β = 0.015, 95% CI: 0.003; 0.028, *p* = 0.017) showed a positive association. No associations were found between saturated and monounsaturated fatty acids and VAI. Conclusions: The EPA + DHA intake, EPA, and DHA intake were inversely associated with VAI in women, but not in men. Omega-6 intake was negatively associated with VAI in men and positively associated with VAI in women. It is important to highlight that, given the cross-sectional design, these associations do not establish temporality or causality.

## 1. Introduction

Obesity and overweight are global health priorities across all life stages, sexes, and most countries [1]. Obesity results from complex and multifactorial determinants, such as lifestyle, biological, and genetic traits [2]. Obesity is often classified by body mass index (BMI) ≥ 30 Kg/m^2^, which shows the imbalance between food intake and energy expenditure [2]. Abnormal and excessive fat accumulation impairs health. It currently affects more than 890 million individuals worldwide [3].

Although obesity involves metabolic, molecular, and genetic events that favor fat accumulation [4,5], the obesogenic environment appears to exert a relevant role [6]. Despite that, not all individuals under an unfavorable environment develop obesity, reinforcing that metabolic response and genetic variations act on individual predisposition to obesity [7]. If not prevented or controlled, obesity induces many comorbidities, such as type 2 diabetes, dyslipidemia, hypertension, chronic kidney disease, non-alcoholic fatty liver disease, and metabolic syndrome, which, when isolated or combined, increase the cardiovascular disease (CVD) risk [7].

Given the serious impact of obesity-related illnesses, adequate monitoring of body weight is crucial. This helps detect overweight and obesity early so treatment can begin. Three-dimensional tomography and magnetic resonance imaging are gold-standard methods for assessing body composition and abdominal obesity. However, due to radiation and high cost, these methods have limited use in clinical practice [8]. Visceral fat accumulation can also be estimated using indirect methods like waist circumference (WC) and its ratios with hip (waist-to-hip ratio—WHR) or height (waist-to-height ratio—WHtR). These measures are now often combined with metabolic parameters to improve sensitivity and specificity. Currently, lipid accumulation product (LAP), Chinese visceral adiposity index (CVAI), BMI metabolic score (BMIm), and visceral adiposity index (VAI) are validated, low-cost tools widely used to monitor obesity and related diseases [8].

VAI is a validated method used to assess visceral adipose tissue and its function according to sex and anthropometric and biochemical parameters, including WC, BMI, triglyceride levels, and high-density lipoprotein cholesterol (HDL-c) [9]. VAI demonstrates greater accuracy compared to the predictive power of WC, BMI, and isolated lipid markers [9]. Over the past decades, several studies have shown that VAI is capable of predicting dysfunctional adipose tissue and also increased cardiometabolic risk in different populations [10,11,12]. Additionally, VAI is also correlated with several adipocytokines, including resistin, leptin, soluble leptin receptors, adiponectin, ghrelin, vascular endothelial growth factor (VEGF), hepatocyte growth factor, tumor necrosis factor alpha (TNF-α), high-sensitivity C-reactive protein (hs-CRP), interleukin (IL)-6, and IL-18, according to Amato et al., in patients with type 2 diabetes [13].

Moreover, visceral adipose tissue dysfunction goes beyond fat accumulation, characterized by impaired insulin signaling and a reduced antilipolytic effect of insulin, leading to increased release of free fatty acids and glycerol into the portal circulation. This continuous flow of free fatty acids contributes to hepatic gluconeogenesis, dyslipidemia, and systemic insulin resistance. In parallel, dysfunctional visceral adipose tissue exhibits increased adrenergic sensitivity, promoting further lipolysis and altered expression of genes related to insulin signaling pathways, such as IRS-2. Furthermore, visceral adipose tissue dysfunction involves increased secretion of proinflammatory cytokines, chemokines, and growth factors, along with reduced adiponectin and increased leptin, chemerin, and resistin, favoring a state of chronic low-grade inflammation. Elevated liver enzymes may also reflect hepatic steatosis secondary to visceral obesity [14].

Diet plays a fundamental role in the prevention and treatment of obesity and CVD, with omega-3 fatty acids being an important target for research [15]. Omega-3 fatty acids are robustly related to improvement in metabolic syndrome, obesity, hypertension, and dyslipidemia, through the reduction in plasma triglycerides (TG) [16], anorexigenic mechanisms [17], and anti-inflammatory and antioxidant roles [18]. Conversely, omega-6 is associated with inflammatory stimulus, with activation of cyclooxygenases and lipoxygenases and consequent generation of prostaglandins, weight gain, and accumulation of visceral fat [19].

Although several benefits have been attributed to omega-3 fatty acids, growing evidence indicates differential effects among their subclasses, with alpha-linolenic acid (ALA) showing weaker associations with cardiovascular risk factors compared with eicosapentaenoic acid (EPA) and docosahexaenoic acid (DHA) [20]. In fact, ALA had no significant effect on inflammatory markers and lipid profile in individuals with obesity or overweight. A prominent response was observed only when taking high doses (≥3.0 g/day) for ≥12 months, suggesting a response dependent on the time and doses [21]. In the insulin resistance mice model, the response to EPA and DHA (1%, 2% and 4%) identified a distinct profile, where DHA (4%) stimulated the browning process by up-regulating peroxisome proliferator-activated receptor gamma (PPAR-γ), while EPA (4%) showed an anti-obesity effect independent of PPAR-γ on obesity [22].

Furthermore, there is a gap in the literature regarding how PUFAs impact CVD-associated risk factors, including visceral adiposity, differently in men and women. In 2023, the Framingham Offspring Cohort study, for example, raised the question of whether divergent associations with cardiometabolic risk in women and men could be associated with differences in dietary fat sources [23]. Also in 2025, a literature review concluded that it is unclear whether there are sex differences in response to the consumption of oily fish or alternative sources of omega-3. However, it reinforces that, in general, the consumption of oily fish should be encouraged in recommendations for women [24].

Therefore, the hypothesis of this study is that dietary intake of omega-3 fatty acids is inversely associated with visceral fat deposition and dysfunction, and omega-6 is positively associated with these alterations in a large populational study. To assess this, we investigated whether habitual fatty acid intake is associated with visceral fat deposition and dysfunction, estimated by VAI in participants of a population-based cross-sectional study. Additionally, we tested whether these associations were dependent on the type and amount of fatty acid intake.

## 2. Results

A total of 697 individuals were included in the study, of whom 51.5% were women (*n* = 399). The participants had a mean age of 43.6 ± 14.5 years and a mean BMI of 28.76 ± 5.58 kg/m^2^, consistent with overweight. High VAI was associated with older individuals and high prevalence of smokers (*p* < 0.001 for all) in both sexes. However, the prevalence of sedentary individuals was lower in those with higher VAI. Regarding current diseases, the group with high VAI had high prevalence of hypertension, diabetes mellitus, dyslipidemia, resulting in increased CVD risk, also for both sexes (*p* < 0.05 for all) (Table 1).

As expected, individuals with high VAI presented elevate BMI values (Men: 27.29 ± 4.45 vs. 30.37 ± 4.81 kg/m^2^, *p* < 0.001; Women: 26.77 ± 5.35 vs. 30.74 ± 6.33 kg/m^2^, *p* < 0.001), WC (Men: 91.17 ± 10.83 vs. 98.82 ± 11.34 cm, *p* < 0.001; Women: 80.85 ± 11.29 vs. 91.30 ± 13.21 cm, *p* < 0.001), and fat mass (Men: 21.64 ± 5.85 vs. 25.10 ± 6.71%, *p* < 0.001; Women: 33.53 ± 7.17 vs. 39.35 ± 8.51%, *p* < 0.001), evidencing increased total body adiposity (Figure S1).

As shown in Table 2, individuals with higher VAI values presented an overall more adverse metabolic profile, independent of sex. Men and women in the high VAI groups had significantly higher levels of total cholesterol (TC), low-density lipoprotein cholesterol (LDL-c), TG, glucose, and liver enzymes compared with those in the low VAI groups (*p* < 0.05 for all). Conversely, HDL-c concentrations were markedly lower among participants with high VAI.

Table 3 shows the dietary intake of men and women according to VAI categories. Among men, those with higher VAI values had higher total energy intake and higher proportions and absolute amounts of carbohydrates, total lipids, and fatty acids, including SFA, MUFA, PUFA, and their subclasses. Higher mean values of omega-3, EPA, DHA, and the EPA + DHA intake were also observed in this group. Among women, those in the higher VAI category had higher carbohydrate intake and lower intake of protein, total lipids, and their subclasses. Lower mean intakes of SFA, MUFA, PUFA, omega-3, EPA, DHA, and a lower EPA + DHA intake were observed in women with higher VAI.

The linear regression model (Table 4) showed associations between VAI and dietary intake of polyunsaturated fatty acids. In adjusted models (model 2), omega-6 intake was inversely associated with VAI in men. In women, the EPA + DHA intake, EPA, and DHA were negatively associated with VAI, while omega-6 showed a positive association.

Additional analyses were performed to examine the links between VAI and other dietary fatty acid subclasses (Table S1), including SFA and MUFA. No associations were observed between the consumption of SFA, MUFA, and VAI.

## 3. Discussion

In this population-based study with a strong emphasis on the preservation of Germanic culture, our results show that omega-6 intakes were inversely associated with VAI values in men. Furthermore, omega-3, EPA, and DHA were negatively associated with VAI, while omega-6 showed a modest positive association in women. In general, the observed inverse associations between omega-3 fatty acids and VAI are in line with previous evidence suggesting favorable metabolic profiles associated with omega-3 consumption or supplementation [25,26,27,28].

These associations between omega-3 fatty acids and VAI may result from their lipid-lowering effects, as previously described in the literature. Omega-3 fatty acids stimulate β-oxidation, modulating the synthesis and clearance of TG from very low-density lipoprotein (VLDL) particles, in addition to increasing the activity of lipoprotein lipase [29]. Furthermore, omega-3 fatty acids can reduce TG synthesis by modulating the expression of the Sterol Regulatory Element-binding Protein 1c (SREBP-1) and Carbohydrate Response Element-binding Protein (ChREBP) pathways [30]. This issue was investigated by Hao et al. [31], testing the omega-3-to-omega-6 ratio (0.3:1) for 10 weeks. This intervention promoted a significant reduction in body weight and fat mass in obese mice, but when the omega-6 to omega-3 ratio was higher (20:1), a further increase in the body weight of the mice was observed [31]. In another study, it was demonstrated that the administration for 21 weeks of a high-fat diet (45% kcal from fat) containing 7% (*w*/*w*) menhaden oil, providing 4.82% EPA and 3.77% DHA of total fatty acids, decreased adipocyte hypertrophy and reduced TG content. In contrast, a flaxseed oil diet containing 20.81% ALA did not elicit the same effects [20].

In addition, experimental evidence indicates that EPA and DHA can attenuate inflammation through down-regulation of transcription factors, such as nuclear factor kappa B (NF-κB), c-Jun N-terminal kinase (JNK), and NLR family pyrin domain containing 3 (NLRP3), resulting in reduced production of proinflammatory cytokines, including IL-6, IL-1β, IL-18, and TNF-α [32]. Activation of free fatty acid receptors (FFAR1/4) by omega-3 fatty acids contributes to the suppression of these inflammatory pathways and may increase postprandial satiety and reduce food intake [33,34]. Regulation of adipokines, such as leptin and adiponectin, as well as the activation of PPAR-γ, have also been implicated in the improvement of lipid metabolism and energy homeostasis [35,36].

Furthermore, we found divergent sex-related associations between dietary PUFAs intake and VAI. In fact, there is a clear gap in the literature and inconclusive evidence regarding the distinct effects of habitual PUFA intake by sex [28,37,38]. However, existing data indicate that the metabolism and physiological roles of PUFAs differ between men and women, in part due to hormonal regulation and variations in body fat distribution [24]. Women tend to have higher proportions of ALA and DHA and lower proportions of EPA compared to men, particularly before menopause, due to the stimulatory effects of estrogen on the enzymatic conversion of ALA to long-chain n-3 PUFAs through increased desaturase and elongase activity [24]. After menopause, however, the sharp decline in estrogen levels is associated with reduced endogenous synthesis of these fatty acids and unfavorable cardiometabolic changes, such as increased central adiposity, inflammation, and dyslipidemia [24]. Collectively, this evidence supports the hypothesis that hormonal status may explain, in part, the divergent associations observed between PUFA intake and visceral adiposity in men and women.

Another relevant point to consider is the influence of whole-food dietary patterns on visceral adiposity. Although our analysis focused on individual and grouped fatty acids, it is clear that other dietary components interact synergistically with adiposity and related anthropometric indicators. Several studies corroborate this view, including Ferguson et al. [39], who found an inverse association between adherence to the DASH diet and VAI among older adults; Xu et al. [40] using decision tree modeling based on NHANES data identified VAI risk, protein intake, and fiber intake as key predictors of cardiovascular disease; and Nazari et al. [41] observed a marginal inverse association between the Lifelines Diet Score and VAI in Iranian women. Furthermore, Moslehi et al. [42] highlighted the relevance of macronutrient substitution models in understanding the metabolic impact of dietary fat quality.

In addition, a dietary source of fatty acids can also influence the effects on lipid metabolism and fat accumulation. It is known that the Brazilian population consumes a high amount of red meat [43]. In particular, individuals living in the southern region of Brazil have high meat consumption (both processed and fresh), with men consuming more meat than women [44]. In this cohort, oleic acid likely derives predominantly from animal sources (e.g., meat—processed and unprocessed and dairy fats) and, to a lesser extent, from plant oils; source-specific effects should be considered when interpreting MUFA associations. Taken together, these findings suggest that the impact of fatty acids on visceral adiposity cannot be fully understood in isolation. Future studies should incorporate comprehensive analyses of dietary patterns to contextualize the role of fatty acids within the complexity of real-world eating behaviors. Regarding this issue, all fatty acids were adjusted by energy, and regression models considered the potential interaction with palmitic, stearic, and oleic fatty acids, besides other confounders.

The results of the present study should be interpreted, taking into account some limitations. First, this is a cross-sectional study, in which it is not possible to infer a causal relationship from the data presented. In addition, the FFQ used for dietary assessment presents inherent limitations. Because it relies on the participant’s memory and perception, FFQs are subject to recall bias and systematic underreporting or overreporting of food intake, particularly among overweight or obese individuals. Moreover, FFQs have limited precision for estimating the absolute intake of specific nutrients, particularly individual fatty acid subclasses. To minimize these potential biases, all interviews were conducted by trained dietitians, and questionnaires with incomplete or inconsistent responses were excluded from the analyses. Despite these limitations, the FFQ applied in this study was previously developed and validated for the Brazilian population [45] and adapted for the population enrolled [46]; however, it was not specifically validated for the estimation of fatty acid subclasses, which should be considered when interpreting the results. Additionally, we cannot discharge the influence of genetic factors not investigated here, but recent evidence shows that Caveolin-1 polymorphisms may interact with dietary fat quality to influence adiposity, suggesting gene-diet interactions play a role in metabolic responses [47].

Another limitation is that, although VAI is a validated and practical surrogate marker based on anthropometric and lipid parameters, it cannot fully capture the complex metabolic activity of visceral fat. Therefore, more accurate and sophisticated techniques, such as computed tomography or magnetic resonance imaging, provide direct quantification of visceral adipose tissue, but are expensive, time-consuming, and impractical in large-scale population studies. In this context, the inclusion of liver enzyme analyses in our results partially offsets this limitation, offering additional insights into metabolic disorders associated with visceral adipose tissue dysfunction. However, the lack of data on insulin resistance, lipokines, and inflammatory biomarkers limits a more comprehensive assessment of adipose tissue dysfunction and its systemic metabolic implications.

Overall, the findings indicate that the relationship between PUFAs and VAI is multifaceted and depends on both the quality and composition of dietary fats. The sex-related differences observed in these associations underscore the importance of considering biological and behavioral factors that modulate fatty acid metabolism. It is essential to note that the present study contributes to the current body of evidence by emphasizing the relevance of sex-specific recommendations regarding PUFA consumption and metabolism. Further prospective and interventional studies are warranted to confirm these observations and to clarify whether such variations stem from intrinsic biological mechanisms or are shaped by context-dependent dietary and lifestyle patterns.

## 4. Materials and Methods

### 4.1. Sample and Study Design

**Figure 1:**
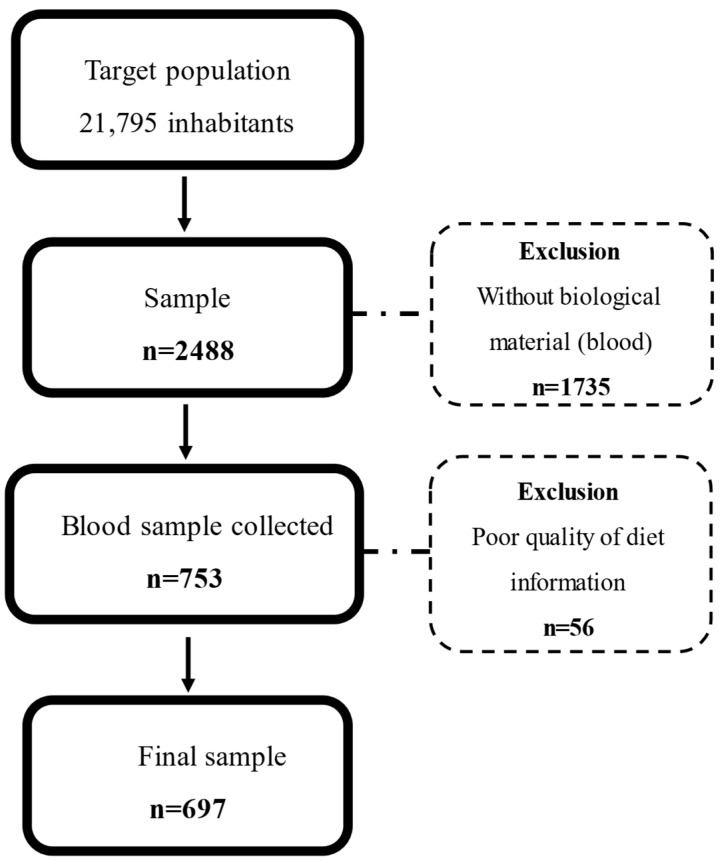
Study flowchart.

This is a cross-sectional study using baseline data from the SHIP-Brazil study. The sample size calculation is based on a 50% event prevalence and 5% precision, estimating a total of 3678 individuals from 21,795 inhabitants of Pomerode in 2010. Accounting for losses and refusals during the baseline data collection, the final sample was 2488 participants, of whom 697 individuals fulfilled all data for this study (Figure 1). Details of the sample size calculation and design study were previously described by Santa Helena et al. [48].

This study included individuals of both sexes, aged between 20 and 79 years, residing in Pomerode, Santa Catarina, Brazil, for at least 6 months. This city is characterized by intense preservation of Germanic culture as a result of the migration movement to Brazil that started in the second half of the 19th century. Individuals with physical, cognitive, or mental disabilities that did not allow them to answer the questionnaires or move to the Research Center, individuals who did not speak Portuguese, as well as malnourished individuals, pregnant and lactating women, participants in clinical intervention protocols, and users of illicit drugs were excluded from this study [48].

This study was approved by the Ethics Committee for Research with Human (No. 8903924.0.0000.5421), and all data collection procedures are described in the protocols of the SHIP-Brazil cohort and can be accessed at https://www.estudoshipbrazil.com.br/pesquisadores (accessed on 1 July 2024).

### 4.2. Sociodemographic Characterizations

Information was collected with structured questionnaires through direct interviews on the following variables: age; sex; race; level of physical activity using the International Physical Activity Questionnaire (IPAQ) questionnaire and classified in walking, moderate/vigorous physical activity or sedentary [49]; smoking; risk of alcohol abuse was classified in low risk versus high risk—moderate/high/severe [50]; current diseases, including self-reported T2D and dyslipidemia. Hypertension was measured by proxy variable—Systolic blood pressure (SBP) ≥ 140 mmHg and/or diastolic (DBP) blood pressure ≥ 90 mmHg and/or use of antihypertensive medication and/or medical diagnosis. SBP and DBP blood pressure were measured with OMRON705-IT devices, validated according to the guidelines of the British Hypertension Society (BHS) [51].

### 4.3. Food Consumption Analysis

Data on food consumption were collected using an adapted food frequency questionnaire (FFQ), administered by trained and qualified interviewers. The FFQ consisted of 118 food items, and habitual consumption (quantitatively and qualitatively) was based on a previously validated questionnaire for the Brazilian population [45]. The nutritional composition and home measurements of regional recipes were obtained through technical sheets, with ingredients known and described by local residents [52]. This process included the following typical foods: bread fried in lard, bread with sardines and egg, apfelstrudel, cat ear, duck or stuffed duck, sweet potato gnocchi, meat dumpling, white black pudding (leberwurst) and dark (blutwurst), butter biscuit, homemade mayonnaise, colonial cheese, kochkäse, pork fat, colonial milk, bovine tongue, cuka cake, pork head jelly (sülse), cream, duck blood soup (schwarzsauer), loquat (plum), and draft beer. Traditional Brazilian foods, such as cajá, winged yam, yam, cooked plantain, cashew, small crab, crab, acarajé, Bahia food (vatapá, caruru, and fish moqueca), chicory, okra, pistachio, cottage cheese, buffalo mozzarella, polengui cheese, and flatbread, were excluded [52]. All data collected underwent quality control, with verification of missing data, duplication, and analysis of inconsistencies [44].

Total calories, carbohydrates, proteins, lipids, total SFA, palmitic acid, stearic acid, total monounsaturated fatty acids (MUFAs), oleic acid, total polyunsaturated fatty acids (PUFAs), omega-6 (linoleic, dihomo-γ-linolenic, and arachidonic acids), and omega-3 (ALA, stearidonic acid, EPA, DPA, DHA) were assessed using the Food Processor program, version 10.11.0 (ESHA Research, Salem, OR, USA). The dietary data were analyzed using the Food Processor Nutrition Analysis Software, version 10.11.0 (ESHA Research, Salem, OR, USA). Dietary data were analyzed using the USDA National Nutrient Database, available in the software. To add new processed foods, we used the manufacturer’s labels. For typical Brazilian and German recipes, we entered all ingredients and new foods into the software. Nutrients were adjusted for energy consumption based on the residual method [53]. Extreme consumption values, below the 1st percentile and above the 99th percentile, were considered outliers and not included in the analyses. From the content of EPA and DHA was calculated the EPA + DHA intake that consists of the sum of these fatty acids.

### 4.4. Anthropometric Assessment and Body Composition

Anthropometric measurements were obtained by trained evaluators following standardized protocols, https://www.estudoshipbrazil.com.br/pesquisadores (accessed on 1 July 2024). Height and weight were measured using a stadiometer coupled to an electronic scale (W300, WELMY^®^, Santa Bárbara d’Oeste, Brazil), with participants barefoot and standing upright. BMI was calculated as weight (kg) divided by height squared (m^2^) and classified according to the WHO criteria for adults [54] and the PAHO criteria for older adults [55]. WC was measured midway between the lowest rib and the iliac crest, using an inelastic tape, after normal expiration. Body fat percentage was estimated by bioelectrical impedance analysis (Biodynamics 450, TBW, São Paulo, Brazil) with electrodes placed on the dominant limb. Standard pre-assessment procedures were followed, including the removal of metal accessories.

The VAI was calculated using the validated sex-specific equation proposed by Amato et al. [9]:

Men VAI = (CC/39.68 + (1.88 × BMI)) × (TG/1.03) × (1.31/HDL-c)

Women VAI = (CC/36.58 + (1.89 × BMI)) × (TG/0.81) × (1.52/HDL-c)

### 4.5. Estimate of Cardiovascular Disease Risk

Cardiovascular disease risk was estimated using the predictive pooled cohort equations (PCE) proposed by the American College of Cardiology/American Heart of Cardiology (ACC/AHA). This equation considers the following variables: sex (men and women), age (40 to 79 years), race (white and African-American), SBP (90–200 mmHg), DBP (60–130 mmHg), HDL-c (20–100 mg/dL), total cholesterol (TC) (130–320 mg/dL), diagnosis of diabetes mellitus, use of antihypertensive drugs, and smoking (yes and no) [56]. Based on the equation, individuals are classified according to the chance to have a major cardiovascular (MACE) event in 10 years: low CVD risk (<5%); borderline CVD risk (≥5% to 7.4%); intermediate CVD risk (≥7.5% to 19.9%); and high CVD risk (≥20%), https://tools.acc.org/ascvd-risk-estimator-plus/#!/calculate/estimate/ (accessed on 15 October 2024). In this study, participants were grouped into low CVD risk (<7.5%) and high CVD risk (≥7.5%).

### 4.6. Biochemical Analyses

After a 10 to 12 h fasting, blood samples (20.0 mL) were collected using vacutainer tubes containing ethylenediaminetetraacetic acid (EDTA) (1.0 mg/mL) to obtain plasma (3000 rpm, 15 min, 4 °C). From plasma, TC, TG, and HDL-c concentrations were assessed using enzymatic and colorimetric reagents (Labtest, Lagoa Santa, Brazil). The low-density lipoprotein (LDL-c) was determined using the Friedewald formula [57], where LDL-c = TC−HDL-c−TG/5. Plasma glucose determination was performed according to the manufacturer’s instructions, using the commercial enzymatic and colorimetric kit Glucose PAP Liquiform (Labtest^®^, São Paulo, Brazil). All parameters were analyzed in the semi-automated Cobas Mira system (Randox Laboratories Ltd., Crumlin, UK). Liver enzymes, ALT, AST, and GGT, were analyzed using commercial kits according to the manufacturer’s instructions.

### 4.7. Statistical Analyses

All statistical analyses were performed using inverse probability weighting (IPW) to minimize potential selection bias arising from the complex sampling design, following established methodological recommendations [58,59]. This approach allows for participants with lower probabilities of selection to contribute proportionally more to the analyses, thereby reducing bias related to differential inclusion as described by Santa Helena et al. [48].

In this study, the diet variables (SFA, palmitic acid, stearic acid, MUFAs, oleic acid, and PUFAs, omega-6, linoleic, dihomo-γ-linolenic, arachidonic acids, omega-3, ALA, stearidonic acid, EPA, DPA, and DHA) were treated as continuous variables and also categorized into tertiles. In addition, the sample was stratified into two groups, considering the 50th percentile (VAI < p50) and the 50th percentile (VAI ≥ p50), for each sex. The normality of the distribution of continuous variables was assessed using the Kolmogorov–Smirnov test, allowing for the classification of variables as symmetric or asymmetric.

Quantitative variables were described by measures of central tendency (mean) and dispersion (standard deviation) or median (Interquartile Range), while qualitative variables were presented in absolute values (n) and relative frequencies (%). Student’s *t*-test was used to compare symmetric continuous variables, and the Mann–Whitney test was used for asymmetric variables. Differences between proportions were assessed using the chi-square test (χ^2^).

Linear regression models were used to examine the associations between VAI and continuous fat intake variables, stratified by sex. Two models were constructed for the analyses: Model 1 (crude) and Model 2, adjusted for potential confounders, including age, smoking, alcohol consumption, physical activity, Oleic, palmitic, and stearic acids. Regression coefficients (β) and 95% confidence intervals (CI) were estimated for the linear models. To define covariates to add linear regression model, we tested the correlation between EPA, DHA, and omega-3, as well as the EPA + DHA intake and omega-6 and macronutrients, MUFA, SFA, oleic acid, and palmitic acid (Table S2); also, using variance inflation factors (VIF), tolerance values, and the condition index, all lifestyle variables (age, smoking, alcohol consumption, and physical activity) showed VIF values close to 1.0 and low condition indices (<10), indicating no collinearity. Among the fatty acid variables, the condition index reached 24.4 and VIF values up to 10.1, with variance proportions above 0.9 shared among palmitic, stearic, and oleic acids, suggesting high collinearity. Only nutrients without collinearity were added to linear regression models. The level of statistical significance adopted was *p* < 0.05. The analyses were performed using SPSS 23.0 and GraphPad Prism 8.4 software.

## 5. Conclusions

The EPA + DHA intake, EPA, and DHA intake were inversely associated with VAI in women, but not in men. Omega-6 intake was negatively associated with VAI in men and positively associated with VAI in women. These findings highlight the importance of considering sex-specific patterns when evaluating the relationship between dietary fatty acids and visceral adiposity. Future longitudinal and interventional studies are needed to explore causal mechanisms and refine dietary recommendations according to sex-related metabolic responses.

## Figures and Tables

**Table 1:** Sociodemographic, lifestyle, and clinical characteristics of individuals, according to sex and visceral adiposity index (VAI) cut-off points (p50).

Variables	Men (*n* = 298)			Women (*n* = 399)		
	VAI < 3.67 (*n* = 158)	VAI ≥ 3.67 (*n* = 140)	*p*-Value	VAI < 3.80 (*n* = 198)	VAI ≥ 3.80 (*n* = 201)	*p*-Value
Age (years), mean (SD)	42 (15)	43 (13)	<0.001	42 (14)	46 (15)	<0.001
Race, *n* (%)						
White	151 (95.6)	132 (94.3)	0.404	184 (93.4)	191 (6.6)	0.204
Non-white	7 (4.4)	8 (5.7)		13 (6.6)	9 (4.5)	
Physical activity, *n* (%)						
Sedentary	98 (74.7)	94 (67.9)	<0.001	121 (67.8)	118 (57.2)	<0.001
Walking/moderate/vigorous	43 (25.3)	44 (32.1)		59 (32.2)	78 (42.8)	
Smoking status, *n* (%)						
Smoker	9 (5.3)	18 (11)	<0.001	12 (5.1)	17 (6.8)	<0.001
Former smoker	51 (26.1)	35 (15.3)		39 (21.3)	28 (12.4)	
Never	95 (68.6)	85 (73.7)		144 (73.6)	155 (80.7)	
Alcohol consumption, *n* (%)						
Low	92 (59.7)	62 (56.6)	0.338	161 (82.6)	177 (89.4)	0.035
High	77 (40.3)	59 (43.4)		34 (17.4)	21 (10.6)	
Current diseases, *n* (%)						
Hypertension						
No	105 (83.1)	80 (73.8)	<0.001	139 (81.3)	94 (62.2)	<0.001
Yes	44 (16.9)	49 (26.2)		47 (18.7)	88 (37.8)	
Diabetes Mellitus						
No	140 (95.3)	115 (89.2)	<0.001	180 (95.5)	170 (92)	<0.001
Yes	14 (4.7)	20 (10.8)		15 (4.5)	25 (8)	
Dyslipidemia						
No	124 (86.8)	78 (61.2)	<0.001	152 (84)	113 (66)	<0.001
Yes	33 (13.2)	58 (38.8)		44 (16)	83 (34	
CVD risk, *n* (%)						
Low	97 (60.7)	37 (26.4)	<0.001	131 (77)	102 (65)	<0.001
High	61 (39.3)	103 (73.6)		67 (23)	99 (35)	

The cut-off points were based on median (p50) values, by sex. Results are presented as absolute values (n) and frequency (%). Differences between groups were assessed using the chi-square test (χ^2^) for categorical variables. Continuous data are presented as mean and standard deviation, and differences between groups were assessed by the Mann–Whitney test for asymmetric variables. The significant *p*-value adopted was <0.05 for all analyses.

**Table 2:** Biochemical data according to sex and visceral adiposity index (VAI) cut-off points (p50).

Variables	Men (*n* = 298)			Women (*n* = 399)		
	VAI < 3.67 (*n* = 158)	VAI ≥ 3.67 (*n* = 140)	*p*-Value	VAI < 3.80 (*n* = 198)	VAI ≥ 3.80 (*n* = 201)	*p*-Value
Total cholesterol (mg/dL)	171 ± 34.8	201 ± 47.7	<0.001	177 ± 35.7	188 ± 37.5	<0.001
LDL-c (mg/dL)	122 ± 31.4	131 ± 45.8	<0.001	111 ± 32.7	119 ± 33.2	<0.001
HDL-c (mg/dL)	43.6 ± 8.6	32 ± 6.6	<0.001	50 ± 11.1	39 ± 8.9	<0.001
Triglycerides (mg/dL)	83 ± 30.9	193 ± 94.6	<0.001	81 ± 28.2	149 ± 55	<0.001
Glucose (mg/dL)	88 ± 20.9	113 ± 10.6	<0.001	82 ± 21.6	98 ± 29.2	<0.001
ALT (U/L)	12 (10)	16 (13)	<0.001	9 (7)	10 (7)	<0.001
AST (U/L)	20 (7)	23 (8)	<0.001	17 (6)	18 (7)	0.016
GTT (U/L)	22 (17)	29 (19)	<0.001	14 (6)	16 (12)	<0.001

The cut-off points were based on median (p50) values, by sex. Continuous data are presented as mean and standard deviation. Student’s *t*-test was used to compare symmetric continuous variables, and the Mann–Whitney test was used for asymmetric variables. The significant *p*-value adopted was <0.05 for all analyses. Low-density lipoprotein cholesterol (LDL-c); High-density lipoprotein cholesterol (HDL-c); Alanine Aminotransferase (ALT); Aspartate Aminotransferase (AST); Gamma-Glutamyltransferase (GTT).

**Table 3:** Food consumption, according to sex and visceral adiposity index (VAI) cut-off points (p50).

Variables	Men (*n* = 298)			Women (*n* = 399)		
	VAI < 3.67 (*n* = 158)	VAI ≥ 3.67 (*n* = 140)	*p*-Value	VAI < 3.80 (*n* = 198)	VAI ≥ 3.80 (*n* = 201)	*p*-Value
Total calories (kcal)	3409 (843)	3513 (1209)	<0.001	2628 (1237)	2711 (1494)	0.204
Carbohydrates (g)	441 (162)	458 (224)	<0.001	367 (203)	379 (227)	<0.001
Proteins (g)	156 (33)	158 (48)	<0.001	128 (58)	118 (62)	<0.001
Lipids (g)	93 (47)	105 (53)	<0.001	72 (12)	75 (38)	<0.001
SFA (g)	32.6 ± 14.7	34.6 ± 14.1	<0.001	26.5 ± 11.3	25.5 ± 11.6	0.001
Palmitic acid (g)	17.2 ± 7.1	18.8 ± 7.5	<0.001	14.1 ± 5.8	13.8 ± 6.6	<0.001
Stearic acid (g)	7.9 ± 3.2	8.5 ± 3.3	<0.001	6.4 ± 2.6	6 ± 2.5	0.001
MUFA (g)	31.6 ± 12.1	34.9 ± 13.7	<0.001	26.5 ± 11.6	24.7 ± 10.2	<0.001
Oleic acid (g)	1.8 ± 0.8	2 ± 0.9	<0.001	1.6 ± 0.7	1.5 ± 0.6	0.004
PUFA (g)	20.2 ± 8.6	22.6 ± 10.8	<0.001	16.8 ± 8.1	17 ± 9.5	0.047
Omega-6 (g) ‡	15.6 ± 6.1	17 ± 7.8	<0.001	13.2 ± 6.4	12.5 ± 6.2	<0.001
Omega-3 (g) ‡	1.9 ± 0.7	2.1 ± 0.9	<0.001	1.7 ± 0.8	1.6 ± 0.7	0.001
EPA (mg)	63.6 ± 14.1	84.5 ± 18.4	<0.001	89 ± 23.4	49.6 ± 8.1	<0.001
DHA (mg)	129.3 ± 16.8	148.9 ± 14.5	<0.001	125 ± 16.2	90 ± 11.4	<0.001
EPA + DHA intake (mg)	202 ± 29.9	224 ± 21.6	<0.001	200 ± 28.3	150 ± 19.7	<0.001

The cut-off points were based on median (p50) values, by sex. Eicosapentaenoic acid (EPA), Docosahexaenoic acid (DHA). Student’s *t*-test was used to compare symmetric continuous variables, and the Mann–Whitney test was used for asymmetric variables, *p*-value < 0.05. ‡ Omega-6 is the sum of linoleic, dihomo-γ-linolenic, and arachidonic acid; and omega-3 is the sum of ALA, stearidonic acid, EPA, DPA, and DHA.

**Table 4:** Association between visceral adiposity index (VAI) and dietary consumption of polyunsaturated fatty acids.

	Model 1			Model 2		
Men						
**Variables**	**β**	**CI (95%)**	***p*-Value**	**β**	**CI (95%)**	***p*-Value**
Omega-3 (g)	0.001	−0.192; 0.195	0.990	−0.094	−0.307; 0.119	0.338
EPA+DHA intake (mg)	−0.017	−0.356; 0.321	0.921	−0.178	−0.548; 0.193	0.348
EPA (mg)	0.201	−0.508; 0.909	0.579	−0.224	−1.010; 0.563	0.577
DHA (mg)	−0.165	−0.739; 0.410	0.574	−0.369	−0.997; −0.258	0.248
Omega-6 (g)	−0.028	−0.052; −0.003	0.025	−0.033	−0.060; −0.005	0.021
Women						
Omega-3 (g)	−0.305	−0.388; −0.222	<0.001	0.024	−0.056; 0.103	0.564
EPA+DHA intake (mg)	−1.206	−1.479; −0.993	<0.001	−0.396	−0.639; −0.152	0.001
EPA (mg)	−2.768	−3.364; −2.171	<0.001	−0.679	−1.220; −0.138	0.014
DHA (mg)	−2.012	−2.488; −1.537	<0.001	−0.780	−1.207; −0.352	<0.001
Omega-6 (g)	−0.005	−0.18; 0.008	0.457	0.015	0.003; 0.028	0.017

Analyses were conducted by linear regression, stratified by sex, considering the following models: Model 1: crude model. Model 2: adjusted for age, smoking status, alcohol consumption, and physical activity. β: regression coefficient; 95% CI: confidence interval.

## Data Availability

The data presented in this study are available on request from the corresponding author due to ethical reasons.

## Supplementary Materials

The following supporting information can be downloaded at https://www.mdpi.com/article/10.3390/molecules30214245/s1: Figure S1. Anthropometric data, according to sex and Visceral Adiposity Index (VAI); Table S1. Association between visceral adiposity index (VAI) and dietary intake of SAFA and MUFA; Table S2. Correlation table.

## Abbreviations

VAI	visceral adiposity index
BMI	body mass index
CVD	cardiovascular disease
WC	waist circumference
WHtR	waist-to-height
WHR	waist-to-hip ratio
LAP	lipid accumulation product
CVAI	Chinese visceral adiposity index
BMIm	BMI metabolic score
HDL-c	high-density lipoprotein cholesterol
LDL-c	low-density lipoprotein cholesterol
TG	plasma triglycerides
TC	total cholesterol
ALT	Alanine Aminotransferase
AST	Aspartate Aminotransferase
GTT	Gamma-Glutamyltransferase
MUFA	monounsaturated fatty acids
SFA	saturated fatty acids
ALA	alpha-linolenic acid
EPA	eicosapentaenoic acid
