# Supplementary material for: Associations of Omega-3 and Omega-6 Fatty Acids Intake with Visceral Adiposity: Sex-Specific Patterns in a Population-Based Study
Source: Molecules. 2025 Oct 31;30(21):4245. doi: 10.3390/molecules30214245 (PMC12610072; doi:10.3390/molecules30214245)
Supplement: Supplementary file 1 [file molecules-30-04245-s001.zip › molecules-3920072-supplementary.docx]

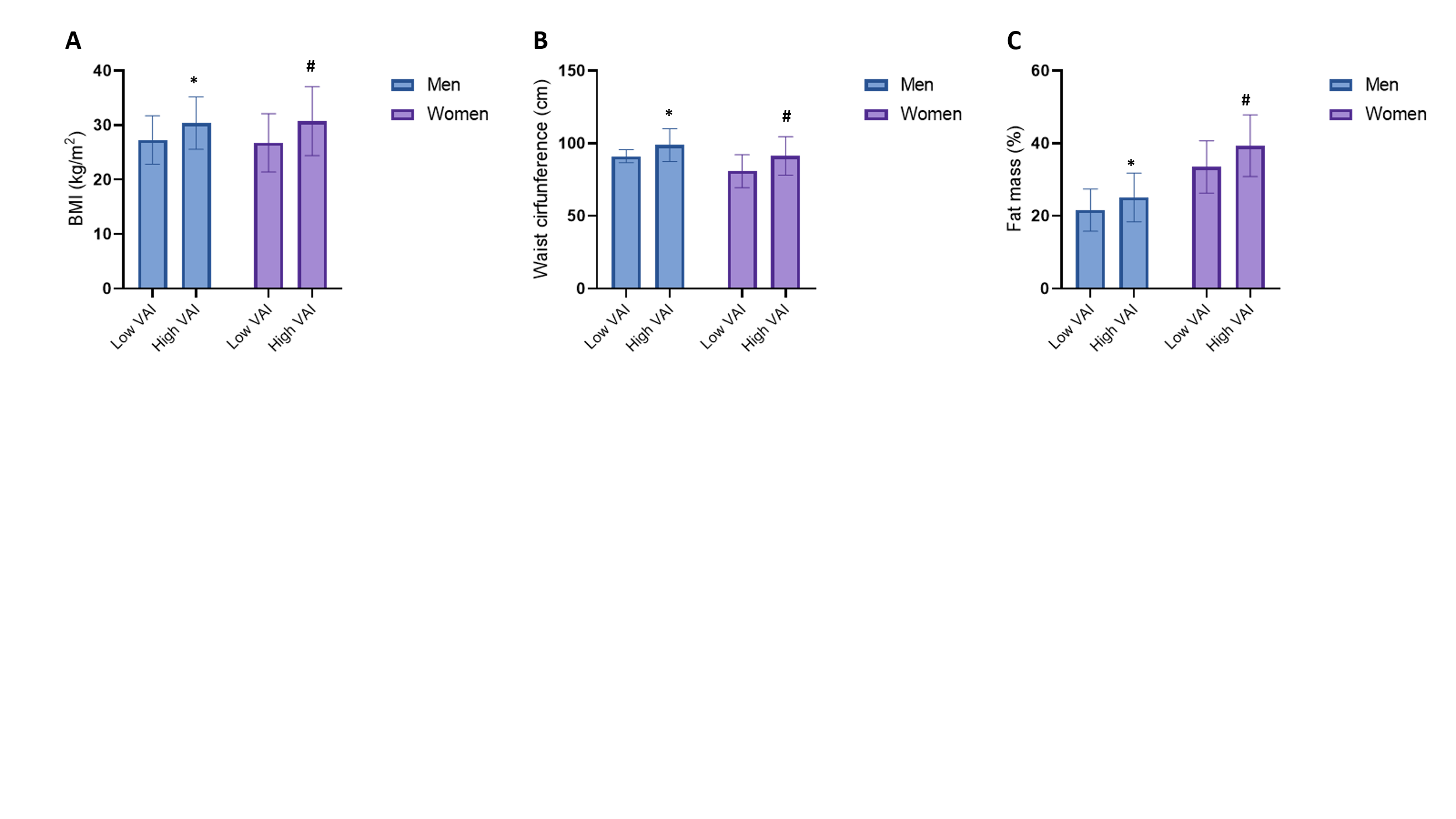


**Figure S1. Anthropometric data, according to sex and Visceral Adiposity Index (VAI).** (A) body mass index (kg/m2); (B) - waist circumference (cm) and (C) fat mass (%) between visceral adiposity index (VAI) groups. Student's t-test was used to compare symmetric continuous variables, and the Mann-Whitney test was used for asymmetric variables, p-value<0.05. *difference between low and high VAI in men; #difference between low and high VAI in women.

**Table S1.** Association between visceral adiposity index (VAI) and dietary intake of SAFA and MUFA.

|  | Model 1 | | | Model 2 | | |
| --- | --- | --- | --- | --- | --- | --- |
| ***Men*** | | | | | | |
| Variable | β | CI (95%) | p-value | β | CI (95%) | p-value |
| SFA (g) | -0.002 | -0.010; 0.006 | 0.577 | 0.000 | -0.010; 0.010 | 0.957 |
| Palmitic acid (g) | 0.008 | -0.009; 0.026 | 0.354 | 0.012 | -0.013; 0.037 | 0.337 |
| Stearic acid (g) | 0.029 | -0.024; 0.081 | 0.283 | 0.050 | -0.018; 0.119 | 0.148 |
| MUFA (g) | 0.002 | -0.010; 0.014 | 0.703 | 0.003 | -0.016; 0.021 | 0.789 |
| Oleic acid (g) | 0.006 | -0.009; 0.021 | 0.422 | 0.009 | -0.014; 0.031 | 0.446 |
| ***Women*** | | | | | | |
| SFA (g) | -0.003 | -0.010; 0.004 | 0.370 | 0.005 | -0.003; 0.013 | 0.186 |
| Palmitic acid (g) | 0.002 | -0.10; 0.14 | 0.757 | 0.014 | -0.002; 0.30 | 0.081 |
| Stearic acid (g) | -0.032 | -0.063; 0.000 | 0.047 | 0.018 | -0.019; 0.054 | 0.345 |
| MUFA (g) | -0.008 | -0.015; -0.001 | 0.018 | 0.003 | -0.005; 0.010 | 0.505 |
| Oleic acid (g) | -0.005 | -0.014; 0.003 | 0.207 | 0.006 | -0.004; 0.015 | 0.230 |

Analyses were conducted by linear regression, stratified by sex, considering the following models: Model 1: crude model. Model 2: adjusted for age, smoking status, alcohol consumption and physical activity. β: regression coefficient; 95% CI: confidence interval.

**Table S2.** Correlation table

|  | Total omega-3 (g/day) | Total omega-6 (g/day) | EPA, mg/day | Docosahexaenoic acid, mg/day | (EPA + DHA) | Carbohydrates (g/day) | Saturated fat (g/day) | Monounsaturated fat (g/day) | Polyunsaturated fat (g/day) | Oleic acid, g/day | Palmitic acid, g/day | Stearic acid g/day |
| --- | --- | --- | --- | --- | --- | --- | --- | --- | --- | --- | --- | --- |
| Total omega-3 (g/day) | 1 | ,794^**^ | ,512^**^ | ,608^**^ | ,588^**^ | ,438^**^ | ,644^**^ | ,741^**^ | ,790^**^ | ,741^**^ | ,673^**^ | ,688^**^ |
| Total omega-6 (g/day) | ,794^**^ | 1 | ,203^**^ | ,264^**^ | ,222^**^ | ,592^**^ | ,594^**^ | ,766^**^ | ,967^**^ | ,789^**^ | ,693^**^ | ,644^**^ |
| EPA, mg/day | ,512^**^ | ,203^**^ | 1 | ,847^**^ | ,948^**^ | 0,011 | ,223^**^ | ,283^**^ | ,287^**^ | ,216^**^ | ,224^**^ | ,188^**^ |
| Docosahexaenoic acid, mg/day | ,608^**^ | ,264^**^ | ,847^**^ | 1 | ,972^**^ | ,027^**^ | ,368^**^ | ,416^**^ | ,309^**^ | ,372^**^ | ,400^**^ | ,340^**^ |
| EPA + DHA | ,588^**^ | ,222^**^ | ,948^**^ | ,972^**^ | 1 | ,034^**^ | ,335^**^ | ,368^**^ | ,283^**^ | ,321^**^ | ,359^**^ | ,305^**^ |
| Carbohydrates (g/day) | ,438^**^ | ,592^**^ | 0,011 | ,027^**^ | ,034^**^ | 1 | ,473^**^ | ,463^**^ | ,621^**^ | ,468^**^ | ,502^**^ | ,454^**^ |
| Saturated fat (g/day) | ,644^**^ | ,594^**^ | ,223^**^ | ,368^**^ | ,335^**^ | ,473^**^ | 1 | ,875^**^ | ,680^**^ | ,847^**^ | ,968^**^ | ,948^**^ |
| Monounsaturated fat (g/day) | ,741^**^ | ,766^**^ | ,283^**^ | ,416^**^ | ,368^**^ | ,463^**^ | ,875^**^ | 1 | ,800^**^ | ,985^**^ | ,904^**^ | ,884^**^ |
| Polyunsaturated fat (g/day) | ,790^**^ | ,967^**^ | ,287^**^ | ,309^**^ | ,283^**^ | ,621^**^ | ,680^**^ | ,800^**^ | 1 | ,797^**^ | ,751^**^ | ,690^**^ |
| Oleic acid, g/day | ,741^**^ | ,789^**^ | ,216^**^ | ,372^**^ | ,321^**^ | ,468^**^ | ,847^**^ | ,985^**^ | ,797^**^ | 1 | ,898^**^ | ,885^**^ |
| Palmitic acid, g/day | ,673^**^ | ,693^**^ | ,224^**^ | ,400^**^ | ,359^**^ | ,502^**^ | ,968^**^ | ,904^**^ | ,751^**^ | ,898^**^ | 1 | ,941^**^ |
| Stearic acid (18:0, g/day) | ,688^**^ | ,644^**^ | ,188^**^ | ,340^**^ | ,305^**^ | ,454^**^ | ,948^**^ | ,884^**^ | ,690^**^ | ,885^**^ | ,941^**^ | 1 |
| ** Significant at the 0.01 level (1 tail). | | | | | | | | | | | | |
| *. Significant at the 0.05 level (1 tail). | | | | | | | | | | | | |
